# Visual and Quantitative Analysis of the Trapping Volume in Dielectrophoresis of Nanoparticles

**DOI:** 10.1021/acs.nanolett.4c02903

**Published:** 2024-08-12

**Authors:** Siarhei Zavatski, Olivier J. F. Martin

**Affiliations:** Nanophotonics and Metrology Laboratory (NAM), Swiss Federal Institute of Technology Lausanne (EPFL), Lausanne 1015, Switzerland

**Keywords:** dielectrophoresis, nanoparticles, force, polarizability, trapping volume, electrokinetic effects

## Abstract

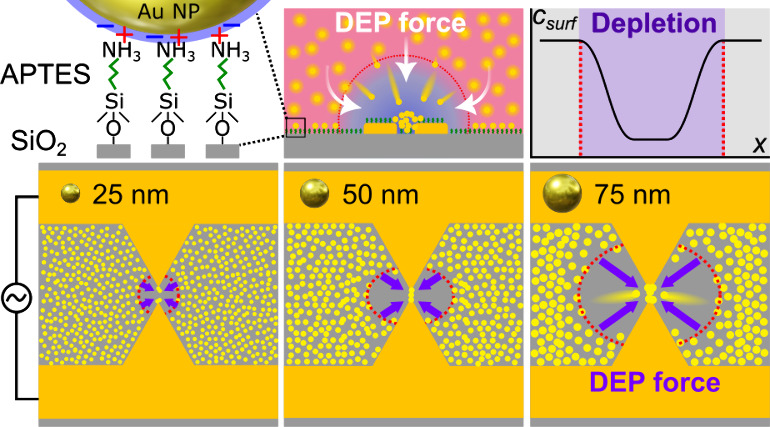

Nanoparticle manipulation requires careful analysis of the forces at play. Unfortunately, traditional force measurement techniques based on the particle velocity do not provide sufficient resolution, while balancing approaches involving counteracting forces are often cumbersome. Here, we demonstrate that a nanoparticle dielectrophoretic response can be quantitatively studied by a straightforward visual delineation of the dielectrophoretic trapping volume. We reveal this volume by detecting the width of the region depleted of gold nanoparticles by the dielectrophoretic force. Comparison of the measured widths for various nanoparticle sizes with numerical simulations obtained by solving the particle-conservation equation shows excellent agreement, thus providing access to the particle physical properties, such as polarizability and size. These findings can be further extended to investigate various types of nano-objects, including bio- and molecular aggregates, and offer a robust characterization tool that can enhance the control of matter at the nanoscale.

Electrokinetic effects enable precise and long-range control of the position of numerous micro- and nanoscale species. As such, they have tremendous potential for both fundamental^1−4^ and applied research.^5−7^ For example, dielectrophoresis (DEP) can renovate the field of separation techniques.^8,9^ Indeed, there is a solid body of research that features the successful utilization of the DEP force for transport,^10^ trapping,^11,12^ separation,^13−15^ and concentration^16−19^ of different inorganic and biological substances. However, a reliable DEP experiment requires a valid experimental estimate of the DEP force, which is usually not straightforward. There is no possibility to measure the DEP force directly and it is typically estimated indirectly, which is possible only as long as a precise theoretical model for DEP exists; unfortunately, this may not always be the case, e.g., for submicron bioparticles.^20−25^ Therefore, developing new force measurement strategies is of fundamental interest for DEP research and its application in the nanosciences.

Several approaches have been proposed to measure the DEP force.^26^ The most common one relies on estimating the particle velocity from videos recorded on an optical microscope.^27−30^ The DEP force can then be determined by solving the Langevin equation.^31,32^ However, a reliable force estimate obtained this way also requires the correct definition of all the other forces that may act on the particle during DEP. Furthermore, if the particles are unlabeled and in low concentration, this method is unsuitable for nanoscale particulates simply because their observation in an optical microscope is challenging. Alternatively, the DEP force can be measured by a balancing approach that requires another counteracting force of known magnitude such that the total force on the target object vanishes. For example, the counteracting force can be optical,^33−35^ gravity,^36^ drag,^13,37,38^ or thermal randomizing caused by the Brownian motion.^39^ We recently used the latter with a gradient array of conductive electrodes to measure the DEP polarizability factors for three proteins.^39^ Unfortunately, the proposed electrodes cannot be utilized to investigate a negative DEP force and the corresponding protein polarizability, because their configuration does not provide clearly defined regions with minimum electric field gradient intensities, where the negative DEP trapping can be detected. Other strategies are also available to measure the DEP force, including measurements of the collection rate,^40−43^ cross–over frequency,^44−46^ and levitation height.^47,48^

Here, we report a straightforward visual representation and quantitative estimate of a particle DEP response that relies on revealing the DEP trapping volume. The key advantage of this technique is that it does not require a special electrode design or complicated experimental setups to gain a quantitative description of the particle movement. Rather, it can be applied to any DEP platform to reveal the interplay between different forces acting on the particle during the experiment. Here, we test the proposed technique in simplified experimental conditions in which the DEP properties of any substance can be determined at a specific electric signal voltage and frequency. The values of this voltage and frequency are chosen such that no net fluid streaming takes place since the side effects of the electric AC field cancel out and only two forces are present in the system: the thermal randomizing and the DEP forces. By introduction of suitable adjustments to the model utilized to analyze the particle concentration distribution after the DEP experiment, it would be possible to extend the proposed technique for more sophisticated experimental conditions. For example, it may be adapted to investigate both negative and positive DEP regimes, thus providing a frequency dependence of the DEP polarizability. Furthermore, it may be used to gain a quantitative understanding of the temperature, pH, and conductivity dependencies of DEP polarizability. All of this can be extremely useful for addressing fundamental challenges in DEP, such as the development and verification of new DEP models for accurate ab initio simulations of the DEP response of bio–nanoparticles.

The DEP platform utilized in this work is depicted in Figure 1a–d (see Materials and Methods in the Supporting Information for fabrication details). It consists of periodically repeated sawtooth gold electrode pairs on a glass substrate separated by a fixed gap of about 4 μm. The lateral distance between sawtooth gaps is 250 μm to avoid any coupling between adjacent electrodes. The DEP is readily observed when these electrodes are immersed in an aqueous dispersion of nanoparticles and energized by an external electrical signal. The time-averaged DEP force acting on a nanoparticle in solution is defined as^8,9^
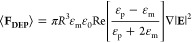
1where *R* is the particle radius, ε_p_ the dielectric constant of the particle, ε_m_ the medium dielectric constant, ε_0_ the vacuum permittivity, and **E** the amplitude of the electric field (not the root-mean-squared electric field).^49^ The term in square brackets in eq 1 is the real part of the Clausius–Mossotti (CM) or DEP polarizability factor—the most critical and intricate parameter for the accurate description of DEP.^20,21^ It not only determines the direction of a particle movement in an inhomogeneous electric field but also influences the magnitude of the DEP force.^9^

**Figure 1:**
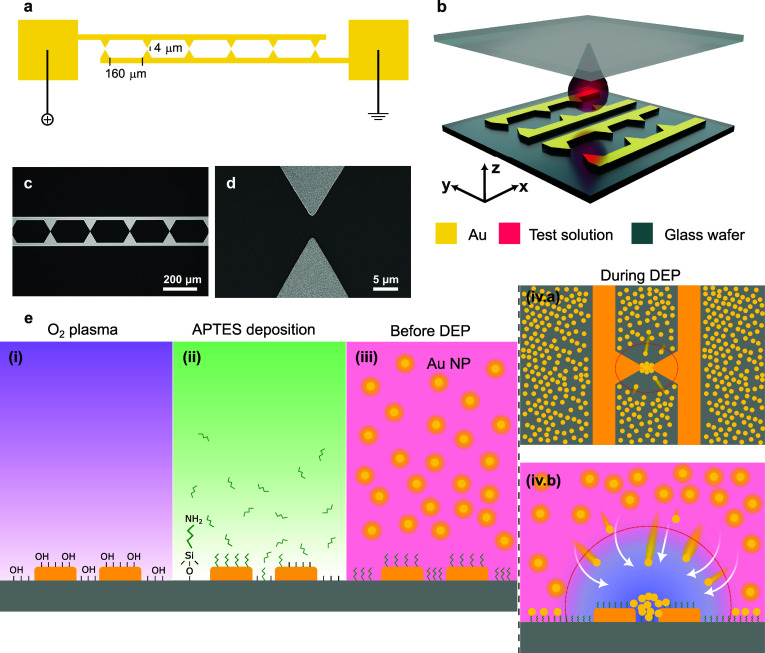
(a) DEP device design showing the unit cell for the sawtooth metal electrode array and (b) schematic representation of the microfluidic chamber utilized for the DEP experiments. (c) Optical microscope and (d) SEM images of a sawtooth metal electrode array (top view). (e) Schematic representation of the DEP device preparation (cross-sectional view) and working principle utilized to visualize the trapping region: (i) DEP device surface cleaning and hydro–oxidation by oxygen plasma treatment; (ii) gas phase (3-aminopropyl)triethoxysilane (APTES) deposition on top of the OH–rich DEP device surface; (iii) the experimental system before AC voltage application, after addition of Au nanoparticles and microfluidic chamber assembly; (iv.a) top and (iv.b) cross-sectional views of the experimental system during the DEP experiment. Au nanoparticles outside the trapping region indicated by the red circle attach to the primary amine (NH_2_–) of APTES molecules through a diffusion–limited process. Au nanoparticles inside the red circle region move toward and accumulate near the sawtooth electrode apexes. This produces two distinct areas on the surface with high and low concentrations, which may be observed by dark-field microscopy.

Our hypothesis to experimentally estimate the DEP parameters in eq 1 is that two distinct volumes must appear near the electrodes during a DEP trapping experiment, with, respectively, high and low concentrations of nanoparticles. The volume with a low concentration—also known as the depletion or trapping volume^50−53^—is where DEP translates nanoparticles away from (negative DEP) or toward (positive DEP) the strongest electric field gradient. This translation occurs because the time-averaged DEP potential energy, ⟨*U*_DEP_⟩, of nanoparticles inside the trapping volume, exceeds the thermal randomizing energy, 3*k*_B_*T*/2:^1,54,55^
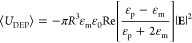
2
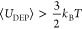
3where *k*_B_ is Boltzmann’s constant and *T* the absolute temperature.

Figure 1e sketches the measurement procedure of the DEP trapping volume. To test our hypothesis, we used Au nanoparticles of different radii, although the technique is applicable to any substance. In this work, we observe only experimentally the cross-section of the trapping volume on the surface recorded by analyzing the dark-field scattering from Au nanoparticles immobilized on the DEP device surface, which is enough to investigate the particle DEP response. To provide an appropriate contrast between high and low (i.e., depleted by DEP) concentration regions, we also functionalize the device with APTES (Figure 1e, steps (i)–(iii)). In the absence of DEP, APTES ensures strong binding of nanoparticles to the surface, producing a uniform nanoparticle layer evidenced by a smooth background scattering intensity. This layer slowly builds up everywhere on the surface by the diffusion-limited motion of nanoparticles, Figure 1e(iii). On the other hand, when the electric field is applied to the electrodes and DEP sets in, nanoparticles are rapidly moved by DEP from within the trapping region to the electrode apexes, preventing interaction with APTES. This leads to a local depletion of the number of nanoparticles adsorbed on the surface, which reduces the dark–field scattering intensity from this region, as illustrated in panels (iv.a) and (iv.b) in Figure 1e. The scattering intensity is recorded and analyzed to obtain its spatial profile.

In general, the concentration of Au nanoparticles in DEP experiments evolves as the result of the interplay between nanoparticle drift and the subsequent diffusion process caused by their DEP-induced redistribution in space. Assuming an ensemble of noninteracting nanoparticles, this concentration profile is given by the particle-conservation equation:^56−58^
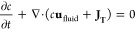
4where *c* = *nV*_p_ is the volume fraction of particles (referred further as the concentration, for brevity) with particle number density *n* and volume *V*_*p*_, **u**_fluid_ is the velocity of the liquid medium, and **J**_**T**_ is the total flux consisting of the sum of the diffusion, **J**_**D**_, sedimentation, **J**_**sedim**_, and DEP fluxes, **J**_**DEP**_:
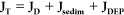
5with
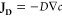
6
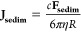
7
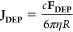
8where *D* = *k*_B_*T*/6πη*R* is the diffusion coefficient for Au nanoparticles, η the liquid viscosity, and **F**_**sedim**_ = (ρ_m_ – ρ_p_)*V*_p_*g* is the sedimentation force with ρ_m_ the medium and ρ_p_ the particle densities, and gravitational acceleration *g*.^1,59^

The solution of eq 4 provides the spatial–temporal evolution of the nanoparticle concentration, which can be effectively compared with experimental results and used to quantitatively characterize the DEP response of a particle. However, obtaining this solution for specific experimental conditions is not straightforward and requires a careful definition of initial and boundary conditions.^57^

In this work, we obtain quantitative information on DEP by comparing the size of the low dark-field intensity measured on the DEP device surface with numerical simulations obtained by solving eq 4 assuming stationary conditions such that the first term on the left–hand side vanishes. This simplification is possible because the experimental conditions are usually long enough to reach equilibrium between the DEP-induced transport and the diffusion of particles. The results obtained by Castellanos et al.^59^ also suggest that we can neglect the sedimentation flux defined in eq 7 because the displacement caused by gravity and buoyancy for particles with a 25–75 nm radius in water is smaller than the displacements induced by DEP and thermal perturbations. Finally, our experimental conditions, including low buffer conductivity (16 μS/cm) and a frequency of the applied electric field (3 MHz), suppress the bulk fluid movement upon DEP, and convection, **u**_fluid_, vanishes. As a result, the nanoparticle concentration on the DEP device surface is proportional to the following solution of eq 4:^1,57^
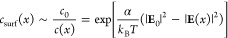
9where *c*_0_ is the initial uniform particle concentration before DEP, , and |**E**_0_|^2^ is the squared magnitude of the electric field on the DEP device surface far from the electrode gap, where it becomes independent of *x*.

The exponential in eq 9 echoes the condition introduced in eq 3 and indicates that the boundaries between depleted and undepleted regions are smeared out for an ensemble of nanoparticles due to their random thermal perturbation.

Equation 9 compares the particle concentrations before and after DEP. In other words, it shows the relation between the number of nanoparticles moving freely by Brownian motion and those translated as a result of applying the DEP force. We can use this equation to simulate the surface concentration profiles observed in experiments for the following reasons. In the absence of the DEP or outside the trapping region, all nanoparticles move freely by thermal randomizing forces associated with Brownian motion. These forces are randomly and equally applied in all directions, meaning that there is a nonzero probability that nanoparticles will hit and be absorbed on the surface. On the other hand, nanoparticles avoid interaction with the surface in the trapping region when DEP is applied because the perpendicular DEP force component, ⟨**F**_**DEP**_ (*z*)⟩, which is responsible for nanoparticle translation to and adsorption onto the surface, is about 200 times weaker that the maximum value of ⟨**F**_**DEP**_ (*x*)⟩ and 18 times weaker than the maximum value of ⟨**F**_**DEP**_ (*y*)⟩ in the gap, which induce nanoparticles’ movement parallel to the surface (see Figure S1 in the Supporting Information for comaprison). Hence, eq 9 effectively shows where the DEP device surface is depleted by nanoparticles, which do not reach it due to translation by the DEP force all the way to the electrodes.

Let us first support the proposed hypothesis and the above analysis with experimental results. Figure 2a–c show the dark-field scattering images acquired for Au nanoparticles with different radii after DEP (15 V_p–p_ and 3 MHz). The regions of high and low dark-field scattering intensities are well visible, with the lowest intensity near the electrode apexes and a progressive intensity increase as one moves away from them. At some distance from the electrode gap, the dark–field intensity reaches a certain magnitude and then remains constant, indicating that one has left the trapped region and entered the undepleted space, where the exponential in eq 9 becomes negligible. Figure 2 also indicates that this transition is observed farther from the electrode gap for larger nanoparticles, which is also expected from eq 9 because the DEP potential energy of nanoparticles has a cubic dependence on their radii. Hence, the obtained scattering profiles can be reliably attributed to generation of the DEP trapping region.

**Figure 2:**
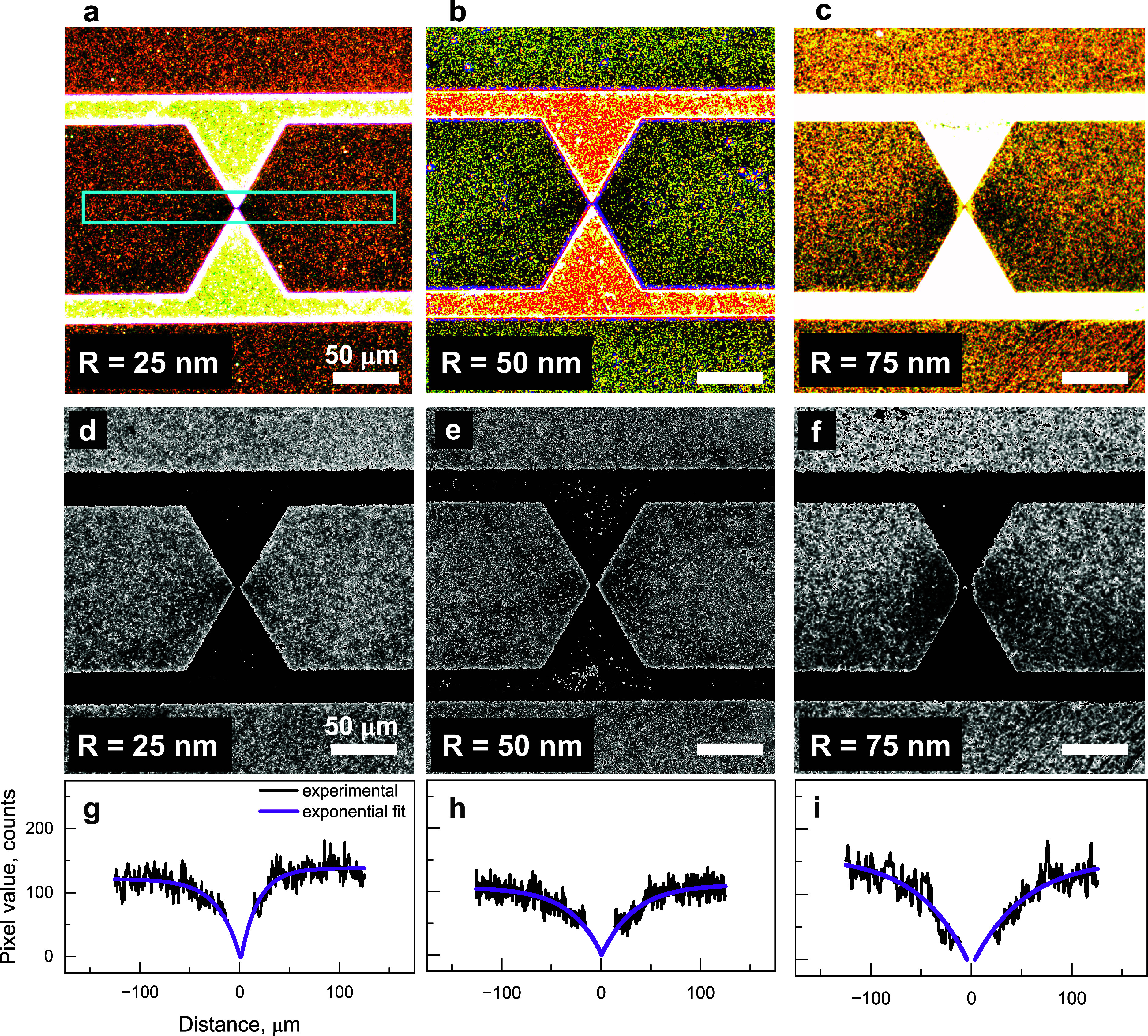
(a–c) Dark–field images acquired for Au nanoparticles with the radius of (a, d) 25 nm, (b, e) 50 nm, and (c, f) 75 nm after DEP at 15 V_p–p_ and 3 MHz. (g–i) Dark–field scattering intensity profiles obtained by integrating within a rectangle shown in cyan in panel (d) (see text for details). An exponential fit, shown in purple, is obtained for the intensity profiles in panels (g–i) after subtracting the data near the electrode gap. All scale bars are 50 μm.

Figure S2 shows examples of dark-field scattering intensity profiles calculated from experimental images depicted in Figure 2a–c. To obtain these profiles, we integrated the scattering intensity in a cyan rectangle, as shown in Figure 2a. The intensity is estimated by taking the average pixel value along the height of the rectangle at each image pixel along the width. We note in Figure S2 a high-intensity scattering peak at the center of the data, which corresponds to the scattering from nanoparticles accumulated by DEP in the highest intensity of the electric field gradient, where the perpendicular DEP force component, ⟨**F**_**DEP**_ (*z*)⟩, is not negligible (see Figure S1 in the Supporting Information). This peak must be omitted in the following procedure because the corresponding region does not intersect with the trapping volume; see Figure 1 e(iv.b). Excluding this peak, we obtain the relevant experimental data shown in black in Figure 2g–i. Next, we perform a fit of these data to obtain the purple profiles shown in Figure 2g–i. Subsequently, the fitted profiles are used to estimate the size of the trapping region by considering the distance from the electrode gap to the middle of the exponential distribution of Au nanoparticles.

Let us now compare the experimental data with the simulated concentration profiles. The 3D simulation domain and corresponding electric field strength distribution near the sawtooth electrode apex are shown in Figure 3a and 3b (see Materials and Methods in the Supporting Information for additional simulation details). This model is based on the effectively fabricated geometry, as shown in Figure 1c,d. The geometrical parameters, including the radii of curvature utilized to simulate the electrode tip apex, were carefully determined using SEM and focused ion beam images.^39^ A maximum electric field strength of 1.72 × 10^7^ V/m was calculated near the electrode apex for an applied peak-to-peak voltage of 15 V.

**Figure 3:**
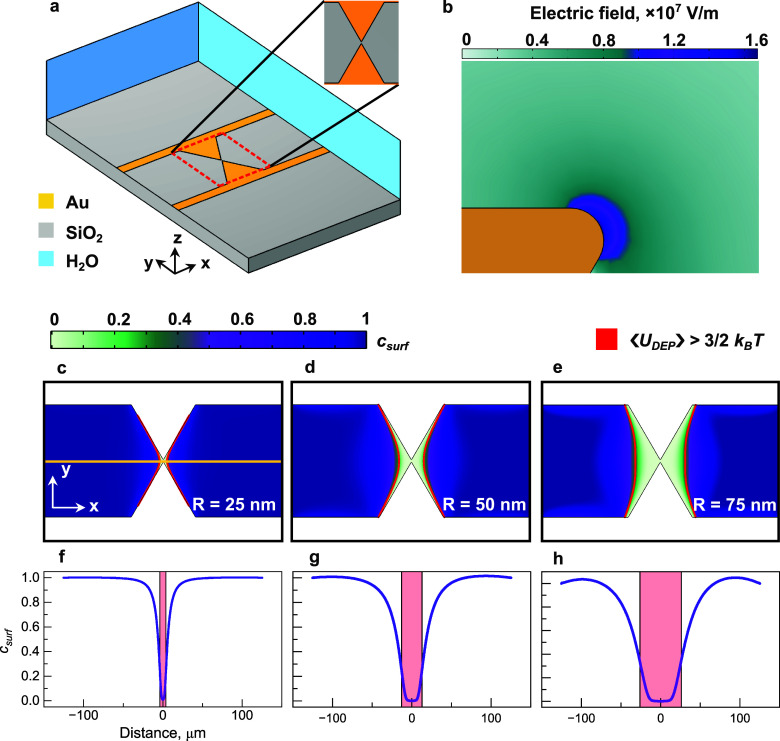
(a) 3D geometry of the DEP device used to simulate (b) the electric field strength distribution near sawtooth metal electrodes. (c–h) 2D simulation results of the concentration distributions for (c, f) 25 nm, (d, g) 50 nm, and (e, h) 75 nm radius Au nanoparticles after applying a sinusoidal electric signal with 15 V_p-p_ peak–to–peak voltage and 3 MHz frequency. The concentration distribution profiles in (f) and (h) were calculated along the yellow line crossing the middle of the gap between adjacent electrode pairs. The red contours in (c–e) and bands in (f–h) depict the area where the DEP potential energy is larger than the thermal diffusion energy, .

We utilize the electric field components **E**_*x*_ and **E**_*y*_ simulated in 3D to compute **J**_**DEP**_ in the plane of the DEP device surface, see eq 8, and calculate the Au nanoparticle concentration distributions, *c*_surf_ (*x*, *y*) by solving eq 4 in 2D (see Materials and Methods in the Supporting Information for additional simulation details that indicate that the same concentration profiles are observed when eq 4 is solved in 3D). The obtained concentration profiles are shown in Figure 3c–h. Figure 3c–e show the spatial variation of the concentration near the electrodes, while Figure 3f–h depict the same concentration profiles along the yellow line in Figure 3c. These figures indicate a significant concentration variation near the electrodes, revealing the shape and size of the trapping regions, which are in good agreement with the experimental scattering profiles shown in Figure 2a–i. The minimum of surface concentration is observed for all the studied nanoparticles in the middle of the gap between the electrodes. It gradually increases with the distance from the gap, approaching the high concentration limit. Besides, Figure 3c–h indicate that the trapping region is wider for larger Au nanoparticle, which is again in agreement with the scaling of ⟨*U*_DEP_⟩ defined by eq 2.

At this point, we should emphasize the importance of considering nanoparticle diffusion to simulate the trapping region size. This can be observed in Figure 3c–h, where the red contour depicts the spatial extension of the condition in eq 3. The width of the trapping region defined by eq 3 and calculated along the yellow line in Figure 3c, varies with the nanoparticle radius: 7.6 μm, 25.9 μm, and 52.4 μm for 25 nm, 50 nm, and 75 nm Au nanoparticles. It is noteworthy that the actual width of the surface concentration variation can be significantly larger than that obtained by balancing the thermal energy, especially for small particle sizes (see the concentration value at which the red band crosses the concentration profile for various Au nanoparticle radii in Figure 3f–h). These results indicate that—when investigating the DEP of a nanoparticle ensemble—the trapping volumes must be estimated by applying the laws of statistical physics.

Let us now compare the obtained experimental scattering profiles to the simulated concentration distributions of Au nanoparticles. Figure 4 shows the dark-field scattering intensity fits for approximately 50 various sawtooth microelectrode pairs. The gray curves represent the corresponding concentration profiles for Au nanoparticles with radii of 25 (Figure 4a), 50 (Figure 4b), and 75 nm (Figure 4c). The purple lines in Figure 4 correspond to the simulated concentration profiles shown in Figure 3f–h. The average experimental sizes of the trapping region after DEP for various nanoparticles are shown in Table 1. These values are in very good agreement with the simulation results.

**Table 1:** Comparison of Experimental and Simulated DEP Trapping Region Sizes, and Corresponding Particle Radii Calculated by Eq (S8)^a^

	Trapping region size, μm		*R*, nm
Au nominal radius, *R*, nm	Experimental	Simulated	Calculated by eq S8
25	16.0 ± 4.4	9.7	30.8 ± 2.9
50	35.6 ± 8.8	36.0	48.5 ± 7.9
75	61.4 ± 12.4	63.7	71.1 ± 10.7

a All experimental values are obtained by analyzing dark–field scattering intensities from approximately 50 electrode pairs.

**Figure 4:**
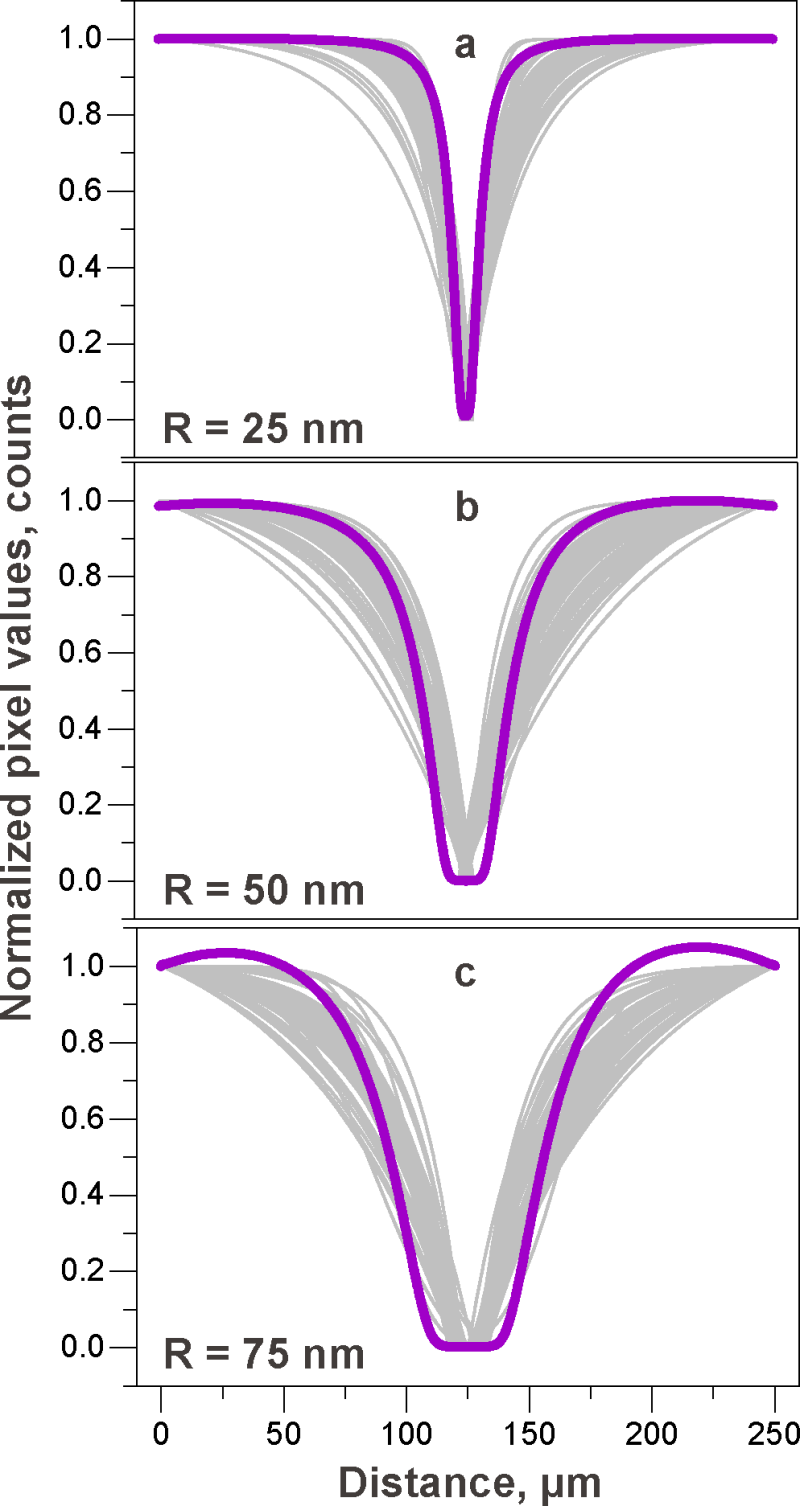
Quantitative analysis of the dark–field scattering intensity profiles acquired from approximately 50 different electrode pairs for (a) 25 nm, (b) 50 nm, and (c) 75 nm Au nanoparticles at 15 V_p–p_ and 3 MHz. The gray lines represent the experimental exponential fits obtained with the procedure outlined in the main text and are similar to those depicted in Figure 2g–i. The purple lines correspond to the simulated concentration profiles shown in Figure 3f–h. Each experimental profile has been normalized between its minimum and its maximum.

To demonstrate that the proposed approach can be effectively utilized for the quantitative characterization of the DEP response of different nanoscopic objects, we analyze the obtained concentration profiles and estimate the Au nanoparticle radius (see the Supporting Information for the calculation procedure). The calculation results are summarized in the last column of Table 1. The agreement between the experimentally deduced radii and their nominal values is excellent. This approach is very general and can be used to determine any parameter in eq (S8), including the DEP polarizability factor.

Let us also note that the accuracy achieved here results from careful choice of the experimental conditions. At least two critical factors may lead to significant errors and must be carefully handled for the correct estimation of the trapping region (see the Supporting Information for further discussion). One relates to the first term in brackets in eq 4 (the convection) and accounts for mass transfer induced by the bulk fluid movement. In very high conductivity media, the convection magnitude may vastly exceed the DEP force, which complicates the trapping region visualization. The second factor that may obscure the trapping region in experiments is an inappropriate choice of the DEP electrode geometry. Indeed, the electrodes must ensure sufficient space between adjacent DEP traps to prevent the intersection of their trapping regions.

In summary, we have demonstrated that experimental visualization of the equilibrium between particle diffusion and DEP translation can be utilized to investigate the DEP response of nanoscopic objects. As an example, we chose a colloidal solution of Au nanoparticles with different radii, calculated their trapping volumes from the concentration profiles measured in experiments, and compared the obtained results with numerical simulations. We also utilized these experimental concentration profiles to extract quantitative information on the system under test, here the Au nanoparticle radii. An excellent agreement was found between simulations and experiments, indicating the robustness of the proposed technique, which can be useful to investigate a broad diversity of analytes for which more sophisticated DEP models are required.

As an outlook, let us mention that the demonstrated approach to produce detectable concentration profiles of the analyte on the DEP device surface can be used to investigate other objects using an appropriate surface linker. For many interesting analytes, the technique can be applied without significant modifications. For example, proteins can also attach to an APTES-functionalized surface,^60^ similar to the Au nanoparticles utilized in the present study. Therefore, the proposed method can be extremely useful for investigating protein DEP, where the classical DEP models predict a much stronger DEP force required for protein trapping than that revealed in experiments.^13,39,54,61−63^ This method can be used to measure quantitative information on protein DEP and thus verify new theoretical models.^22−25,64^ Overall, it will render DEP a more quantitative and versatile tool for manipulations at the micro- and nanoscales.

## Supporting Information Available

Additional details on materials and methods utilized in this study. Additional discussion on the effect of various factors that may lead to significant errors and must be carefully handled for the correct estimation of the trapping region. Additional simulation results showing *x*-, *y*- and *z*-components of the DEP force in the plane of the DEP device surface. (Figure S1). Additional dark–field scattering intensity profiles obtained for raw experimental images indicated in Figure 2a–c in the main text (Figure S2). Additional simulation results of the concentration distributions of Au nanoparticles in 3D simulation domain (Figure S3). Comparison of concentration profiles of 25, 50, and 75 nm Au nanoparticles obtained by numerical simulations and calculations using eq (9) (Figure S4). Additional simulation results for sawtooth metal electrodes showing the magnitude of the electric field squared in the plane of the DEP device surface (Figure S5). Additional dark-field images acquired after DEP at 15 V and 500 kHz (Figure S6). Additional simulation results for sawtooth metal electrodes placed 83.5 μm apart from each other (Figure S7) (PDF)
DEP movement of Au nanoparticles near sawtooth electrodes at 15 V_p-p_ and 500 kHz (Video S1) (MP4)
DEP movement of Au nanoparticles near sawtooth electrodes at 15 V_p-p_ and 3 MHz (Video S2) (MP4)
