# Supplementary material for: Visual and Quantitative Analysis of the Trapping Volume in Dielectrophoresis of Nanoparticles
Source: Nano Lett. 2024 Aug 12;24(33):10305–12. doi: 10.1021/acs.nanolett.4c02903 (PMC11342383; doi:10.1021/acs.nanolett.4c02903)
Supplement: Supplementary file 1 — nl4c02903_si_001.pdf [file nl4c02903_si_001.pdf]

Supporting Information for:

VISUAL AND QUANTITATIVE ANALYSIS OF THE TRAPPING VOLUME IN DIELECTROPHORESIS OF NANOPARTICLES

Siarhei Zavatski and Olivier J.F. Martin**

Nanophotonics and Metrology Laboratory (NAM), Swiss Federal Institute of Technology Lausanne (EPFL), Lausanne 1015, Switzerland

E-mail: siarhei.zavatski@epfl.ch; olivier.martin@epfl.ch

Materials and Methods

Chemicals. (3–Aminopropyl)triethoxysilane (APTES, 99 %), acetone, 2–propanol (IPA, ≥99.5 %), ethanol (≥99 %), toluene (anhydrous, ≥99.8 %), 50 nm, 100 nm, and 150 nm diameter gold nanoparticles (NPs, stabilized suspension in citrate buffer) were purchased from Sigma–Aldrich. 0.22 μm syringe filters with polytetrafluorethylen (PTFE) membrane were obtained from Whatman Anotop. 9 mm diameter imaging spacers were acquired from Grace Bio–Labs SecureSeal.

Dielectrophoretic device fabrication. Standard microelectronics techniques were employed for the dielectrophoretic device fabrication,[S1,S2] and the detailed procedure is described elsewhere.[S3] Briefly, a 100 mm borosilicate wafer was cleaned in piranha solution and treated in high–frequency oxygen plasma. Standard photolithography procedures, including negative photoresist spin–coating, exposure, and development, were utilized to produce sawtooth microelectrode patterns on a borosilicate wafer. Next, the electron beam evaporation technique was used to deposit 5 nm Ti and 100 nm Au electrode material. Finally, the lift–off step and wafer dicing were conducted to produce 31 x 25 mm^2 DEP chips.

Numerical simulations. The finite–elements simulations for the developed DEP device were performed using the AC/DC and Mathematics (classical partial differential equations, stabilized convection-diffusion equation) modules of COMSOL Multiphysics 6.1. The electric field intensity distribution was calculated near sawtooth metal electrodes based on the simulation of the potential ϕ distribution by solving the Laplace equation: $\nabla^2\phi = 0$. The electric field thus was obtained as $\mathbf{E} = -\nabla\phi$. Simulations were conducted for a unit cell of one gold sawtooth electrode pair attached to the 20 μm wide gold rectangle. The thickness of electrodes and the gap size between sawtooth

pairs were 100 nm and 4.1 μm, respectively. Electrodes were located on 250.4 × 585.43 × 30 μm (width × depth × height) SiO_2 substrate and immersed in the water medium of the same width and depth but with 120 μm height. One of the electrodes from the pair was grounded, while the boundary condition for the second was $\phi = V_{p-p} / 2$, where $V_{p-p} = 15$ V, which corresponds to the experimental value of the electric field. The frequency of the electric field was 3 MHz. Periodic boundaries were applied in $\pm x$ direction, and electric insulation conditions were for the remaining boundaries. The field was simulated in a water background, assuming a dielectric permittivity ε_m = 78 and an electrical conductivity σ_m of 16 μS/cm. Gold with σ_{gold} = 456 kS/cm and ε_{gold} = 6.9 was used for the electrode material. The minimum mesh size used for the discretization was 5 nm near the electrode apex, which was found in our previous work to provide a nearly constant electric field value (independent of the mesh) within a reasonable computation time (see Ref. 39 in the main text).

The Au NPs concentration distribution was calculated in 2D in the plane of the DEP device surface by solving the modified particle-conservation equation (see Eq. (4) in the main text) to account for the particle steric effect.[S4] This modification is important to improve the convergence of the simulation results and obtain realistic particle concentrations. No flux boundary conditions were applied anywhere except the left and right boundaries of the simulation domain, for which Dirichlet boundary conditions were introduced, such that the particle volume fraction was kept constant, $c = 0.001$. The initial volume fraction of Au nanoparticles in a simulation domain was also $c_0 = 0.001$. The minimum mesh size used for the discretization in this case was 5.6 nm in the gap between electrodes. The particle transport was simulated in a water background, assuming the same physical properties as at the electric field simulation step. The solution was obtained for a stationary condition such that the first term in Eq. (4) was set to zero.

The following parameters were taken for solving Eq. (4) in COMSOL: $R = 25$ nm, 50 nm, 75 nm, $D = k_B T / 6\pi\eta R$ with $\eta = 8.9 \cdot 10^{-4}$ Pa·s, T = 300 K, and $|\mathbf{E}(x, y, z)|$ obtained by 3D COMSOL simulations.

APTES functionalization of DEP device surface. All DEP chips fabricated in this work were functionalized by APTES via a gas-phase deposition process. First, a DEP chip was thoroughly washed in acetone, IPA, and deionized water by placing it in an ultrasonic bath, followed by drying with N_2. Next, the chip was treated in a high-frequency oxygen plasma (Tepla 300) at 1000 W for 5 min with 400 ml/min O_2 flow. After that, the chip was rewashed with ethanol, deionized water, and dried with N_2. Immediately after cleaning and surface activation, the DEP chip was placed inside a glass beaker together with 1 mL of 99 % APTES, which was isolated in an opened vial to ensure efficient evaporation and prevent direct APTES contact with the chip. Subsequently, the glass beaker was tightly closed and placed in the oven (WTB Binder 7200) for 2 h at 70 °C. After the deposition, the chip was washed with a copious amount of toluene, ethanol, and water to remove unbound APTES residues and dried with N_2. Finally, the APTES–modified DEP chip was placed in a clean glass Petri dish and annealed at 120 °C for 2 h to strengthen the chemical bonds between molecules and the surface.

Characterization. The reproducibility and morphology of the fabricated DEP device were characterized by field-emission SEM (Zeiss MERLIN) managed at 1 kV. The conductivity of the buffer was measured with a commercial conductivity meter (Fisherbrand AE6C Portable Conductimeter). The depletion region visualization was performed by an optical microscope (Leica DM8000) operated in a dark-field mode. Optical microscope (Nikon Optiphot 150) equipped by CCD camera (Chameleon 3 Color Camera, CM3-U3-50S5C-CS) and managed in dark-field mode with a 50× air objective (Nikon, NA = 0.55) was utilized to record all videos.

DEP experiments and postprocessing. All DEP experiments were performed for a colloidal solution of 25 nm, 50 nm, and 75 nm radius Au NPs redispersed in deionized water after double centrifugation for 30 min each at 1100 $\times$ g, 400 $\times$ g, and 300 $\times$ g, respectively. First, the device was energized by the function generator (GW Instek AFG–2125) by applying 15 V peak–to–peak AC voltage at the frequency of 3 MHz for various duration. Next, a drop of freshly prepared Au NPs aqueous solution (14 μL) was placed on top of the DEP device covered with an imaging spacer (Grace Bio–Labs SecureSeal) beforehand. A microscope coverslip was then placed on top of the spacer, avoiding a tight sealing to leave the possibility of removing unbound NPs after DEP, which was done by washing the device with copious amount of deionized water and drying with nitrogen. To record dark-field images of the DEP device after the experiment, the same optical microscope (Leica DM8000) was utilized. All acquired images were subsequently adjusted by image processing software (ImageJ) to enhance contrast and subtract the saturated optical signal and then analyzed to extract depletion region sizes.

Solution of the particle-conservation equation. The particle–conservation equation for an ensemble of non–interacting nanoparticles takes the form of Eq. (4) in the main text. In the steady-state condition, the total particle flux is zero, meaning that $\mathbf{J_T} = 0$ and $\mathbf{J_D} = -\mathbf{J_{DEP}}$ if $\mathbf{J_{sedim}}$ is negligible and any bulk fluid movement is supressed (see Eqs. (5)–(8) and the corresponding discussion in the main text). Besides, we are interested in the particle concentration distribution along the yellow line shown in Figure 3c on the DEP device surface. Therefore, the following equations can be used to find the particle concentration distribution $c(x)$ in one dimension:

$$\mathbf{J_D} = -\mathbf{J_{DEP}}, \tag{S1}$$

$$D\left(\frac{\partial c(x)}{\partial x}\mathbf{i}\right) = \frac{c(x)\mathbf{F_{DEP}}}{6\pi\eta R} = c(x)\frac{\alpha}{6\pi\eta R}\frac{\partial\left|\mathbf{E}(x)\right|^2}{\partial x}\mathbf{i}, \tag{S2}$$

where $\mathbf{i}$ is a unit vector in the transverse x–direction parallel to the DEP–device surface and $\alpha = \pi R^3 \varepsilon_m \varepsilon_0 \operatorname{Re}\left[\frac{\varepsilon_p - \varepsilon_m}{\varepsilon_p + 2\varepsilon_m}\right]$. Therefore,

$$\frac{1}{c(x)}\partial c(x) = \frac{\alpha}{k_B T}\partial\left|\mathbf{E}(x)\right|^2. \tag{S3}$$

Assuming a uniform initial particle distribution c_0 before DEP sets in, we can find

$$\int_{c_0}^{c(x)} \frac{1}{c(x)}\partial c(x) = \frac{\alpha}{k_B T}\int_{\left|\mathbf{E}_0\right|^2}^{\left|\mathbf{E}(x)\right|^2} \partial\left|\mathbf{E}(x)\right|^2, \tag{S4}$$

where $\left|\mathbf{E}_0\right|^2$ is the squared magnitude of the electric field far from the electrode gap, where it is independent of x. Therefore, the solution of Eq. (S4) for the nanoparticle concentration on the DEP device surface takes the following form:

$$c(x) = c_0 \exp\left[\frac{\alpha}{k_B T}\left(\left|\mathbf{E}(x)\right|^2 - \left|\mathbf{E}_0\right|^2\right)\right]. \tag{S5}$$

By rewriting Eq. (S5) to compare the concentration of particles before and after DEP, we obtain:

$$c_{surf}(x) \sim \frac{c_0}{c(x)} = \exp\left[\frac{\alpha}{k_B T}\left(\left|\mathbf{E}_0\right|^2 - \left|\mathbf{E}(x)\right|^2\right)\right], \tag{S6}$$

which is the same as Eq. (9) in the main text.

Quantitative analysis of Au nanoparticle concentrations. We use Eq. (4) in the main text for analyzing the obtained concentration profiles and estimate the Au nanoparticle radius since the agreement between experiments and simulations is excellent. It is convenient to take the ratio of two surface concentrations, $c_{surf}(x_1)$ and $c_{surf}(x_2)$, at two different positions x_1 and x_2 on the line located on the surface (see Figures 3c, S2, and S5) to eliminate the need in defining the absolute surface concentration values:

$$\frac{c_{surf}(x_1)}{c_{surf}(x_2)} = \frac{\exp\left[\frac{\alpha}{k_B T}\left(\left|\mathbf{E}_0\right|^2 - \left|\mathbf{E}(x_1)\right|^2\right)\right]}{\exp\left[\frac{\alpha}{k_B T}\left(\left|\mathbf{E}_0\right|^2 - \left|\mathbf{E}(x_2)\right|^2\right)\right]} = \exp\left[\frac{R^3 B}{k_B T}\left(\left|\mathbf{E}(x_2)\right|^2 - \left|\mathbf{E}(x_1)\right|^2\right)\right], \tag{S7}$$

where $c_{surf}(x_1)$ is the saturated high-value surface concentration obtained along the yellow line at $x_1 = 120\ \mu\text{m}$ from the electrode gap (see Figure 3c in the main text), $c_{surf}(x_2)$ is the concentration obtained along the same yellow line but in the depletion region, such that $c_{surf}(x_2) = c_{surf}(x_1)/2$, and $B = \pi\varepsilon_m\varepsilon_0 \operatorname{Re}\left[\frac{\varepsilon_p - \varepsilon_m}{\varepsilon_p + 2\varepsilon_m}\right]$. Therefore, we can rewrite Eq. (S7) as:

$$R = \left[\frac{k_B T \ln(2)}{B} \frac{1}{\left(|\mathbf{E}(x_2)|^2 - |\mathbf{E}(x_1)|^2\right)}\right]^{1/3}. \tag{S8}$$

It is seen from Eq. (S8) that the nanoparticle radius can be found from the difference of the electric field amplitude squared estimated at the corresponding x coordinates on the surface of the DEP device. We calculate the Au nanoparticle radii by taking the numerically simulated electric field values (see Figure S5) and assuming perfectly polarizable Au spheres $\left(\operatorname{Re}\left[\frac{\varepsilon_p - \varepsilon_m}{\varepsilon_p + 2\varepsilon_m}\right] = 1\right)$, $T = 300$ K, and $\varepsilon_m = 78$.

Choice of the experimental conditions

It is known that applying an electric field to a microfluidic system generates a series of effects on the fluid itself, including electrothermal (ET) flow, electroosmosis and natural convection, which can induce particle movement via the Stokes drag force.[S5] While this drag and other forces such as gravity and buoyancy could influence the measurement and have been discussed in the literature in great detail,[S5,S6–S10] we focus here only on the two electrokinetic effects clearly observed in our experiments – DEP and ET effects – and how to keep them under control.

The ET effect also exists in highly inhomogeneous electric fields and induces two forces acting on a liquid: Coulomb and dielectric forces.[S6,S11–S13] The dominance of one or the other depends on the frequency range and determines the fluid flow direction, since both forces act in opposite directions. Hence, the fluid movement is minimal at the frequency where the transition between these two forces occurs. Besides, the fluid flow velocity determines the magnitude of the drag force for particles. In very high conductivity media, its magnitude may vastly exceed the DEP force, causing particles to follow the direction of the fluid flow. Such a particle behavior would refute our hypothesis that convection can be disregarded in Eq. (4) of the main text and we must ensure that the ET effect is suppressed in our DEP experiments.

The simplest way to suppress fluid flow is to reduce the liquid conductivity and its nonuniform heating to the lowest possible value. In this work, all DEP experiments were performed in water with a low conductivity of 16 μS/cm. Nevertheless, when we applied a sinusoidal signal with the amplitude of $V_{p\text{–}p}$ = 15 V and frequency of 500 kHz, we still observed rapid particle movement that is uncommon for DEP (see Video S1): particles circulated above the electrodes rather than being stably trapped in the gap between them. Detecting the depletion region in these conditions

was impossible (Figure S6). Assuming that the ET effect is responsible for this particle movement, we gradually increased the electric field frequency up to 5 MHz, reaching the transition frequency between Coulomb and dielectric forces, where the fluid movement vanishes and DEP dominates (see Video S2). In this series of experiments, the optimum frequency of 3 MHz was determined, which is in agreement with the ET theory.[S5,S6]

The second important factor that must be considered is the DEP electrode geometry. Indeed, along with generating a strong electric field to build well-discriminated depletion regions, the electrodes must also ensure sufficient space between adjacent DEP traps to prevent overlapping depletion regions. The effect of this intersection can be readily observed in the simulations of our DEP device when the distance between the gaps of two neighboring sawtooth electrode pairs is reduced from 250.4 μm to 83.5 μm (see Figure S7). In this case, the concentration profiles near each electrode pairs merge, which blurs their boundaries. Therefore, one must carefully optimize the electrodes geometry and experimental conditions such that there is a spacing of at least two times the depletion region.

References

[S1] Madou, M. J. *Manufacturing Techniques for Microfabrication and Nanotechnology*; CRC press, **2011**; Vol.2.

[S2] Abasahl, B.; Santschi, C.; Raziman, T. V.; Martin, O. J. F. *Nanotechnology* **2021**, *32*, No. 475202.

[S3] Zavatski, S.; Bandarenka, H.; Martin, O. J. F. *Anal. Chem.* **2023**, *95*, 2958–2966.

[S4] Loucaides, N. G.; Ramos, A.; Georghiou, G. E. *Journal of Electrostatics* **2011**, *69* (2), 111–118.

[S5] Castellanos, A.; Ramos, A.; Gonzalez, A.; Green, N. G.; Morgan, H.; *Journal of Physics D* **2003,** *36* (20), 2584.

[S6] Ramos, A.; Morgan, H.; Green, N. G.; Castellanos, A. *Journal of Physics D: Applied Physics* **1998**, *31* (18), 2338–2353.

[S7] Green, N. G.; Morgan, H.; Milner, J. J. *Journal of Biochemical and Biophysical Methods* **1997**, *35* (2), 89–102.

[S8] Green, N. G.; Ramos, A.; Morgan, H. *Journal of Physics D: Applied Physics* **2000**, *33* (6), 632–641.

[S9] Green, N. G.; Morgan, H. *Journal of Physical Chemistry B* **1999**, *103* (1), 41–50.

[S10] Green, N. G.; Ramos, A.; González, A.; Castellanos, A.; Morgan, H. *Journal of Physics D: Applied Physics* **2000**, *33* (2).

[S11] Green, N. G.; Ramos, A.; González, A.; Castellanos, A.; Morgan, H. *Journal of Electrostatics* **2001**, *53* (2), 71–87.

[S12] Salari, A.; Navi, M.; Lijnse, T.; Dalton, C. *Micromachines* **2019**, *10* (11), 1–27.

[S13] Sun, H.; Ren, Y.; Hou, L.; Tao, Y.; Liu, W.; Jiang, T.; Jiang, H. *Analytical Chemistry* **2019**, *91* (9), 5729–5738.

Supplementary figures

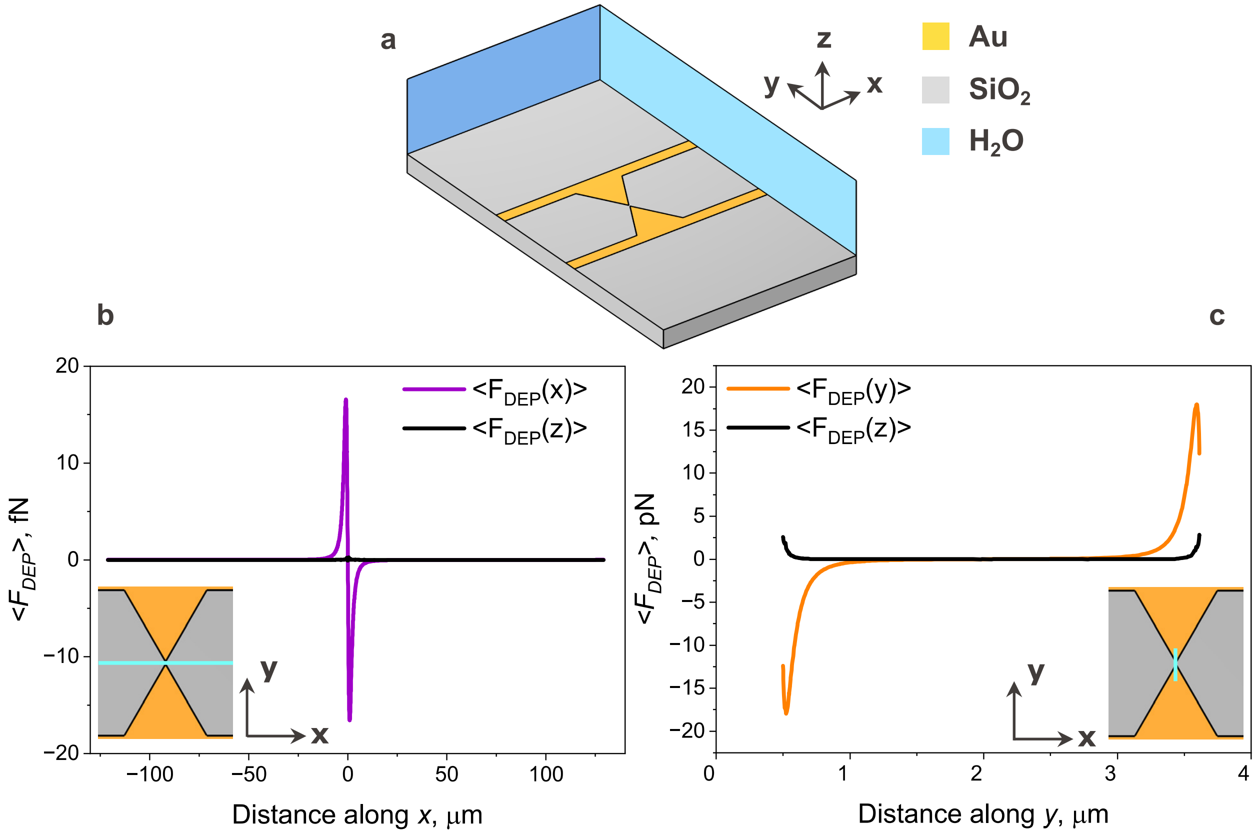

Figure S1. (a) Schematic geometry of the DEP device used to simulate (b) *x*– and *z*–components and (c) *y*– and *z*–components of the DEP force, $\langle \mathbf{F}_{\mathbf{DEP}}(x) \rangle$, $\langle \mathbf{F}_{\mathbf{DEP}}(y) \rangle$, and $\langle \mathbf{F}_{\mathbf{DEP}}(z) \rangle$, along the cyan lines shown in the corresponding insets.

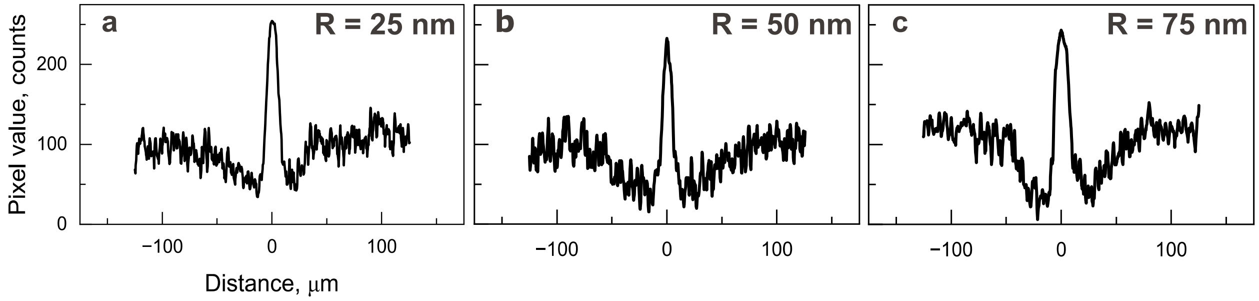

Figure S2. Dark–field scattering intensity profiles obtained for raw experimental images indicated in Fig. 2a–c in the main text.

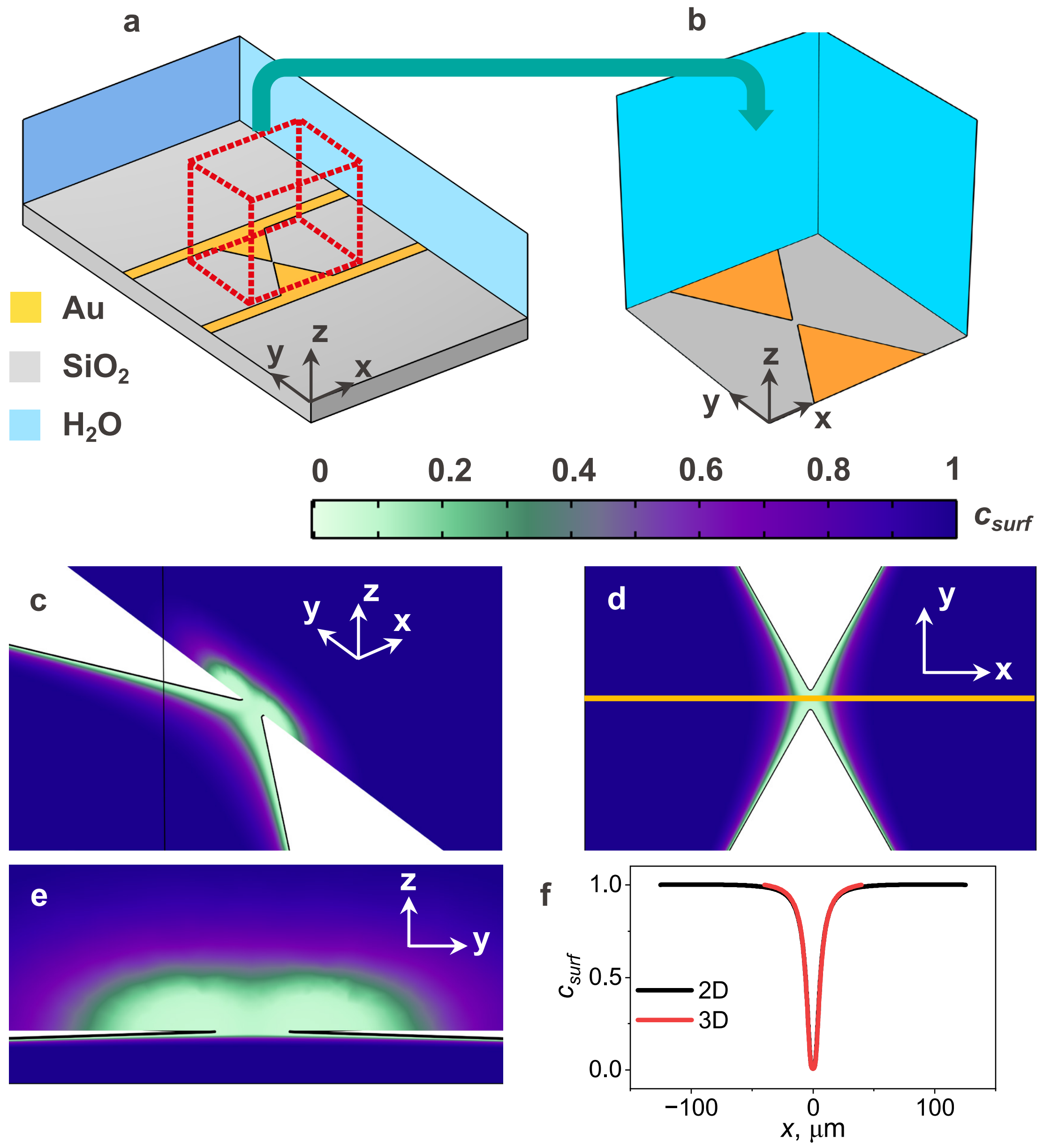

Figure S3. (a) Schematic geometry of the DEP device used to simulate the electric field $\mathbf{E}$, which was transferred to (b) a reduced 3D simulation domain with width × depth × height of 80 × 80 × 80 μm for simulating concentration distribution of Au nanoparticles during DEP. (c) 3D, (d) top and (e) cross–sectional view of the simulation results of the concentration distributions for 25 nm radius Au nanoparticles after applying a sinusoidal electric signal with 15 V peak–to–peak voltage and 3 MHz frequency. (f) The comparison of concentration distribution profiles in 2D and 3D were calculated along the yellow line in (d) crossing the middle of the gap between adjacent electrode pairs.

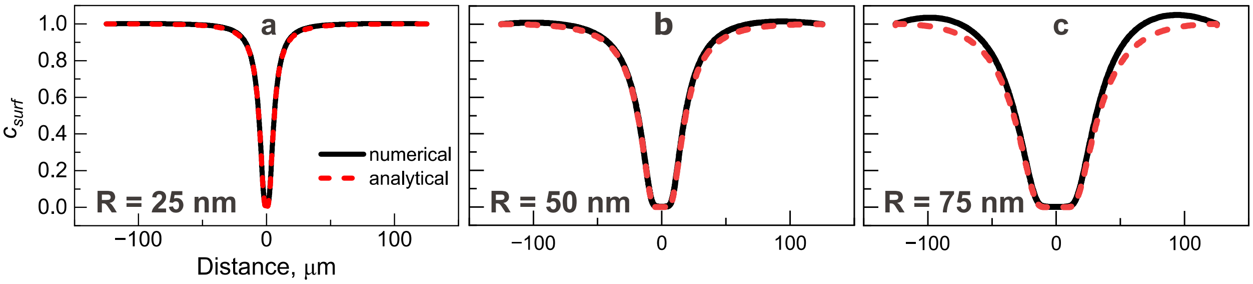

Figure S4. Comparison of concentration profiles of (a) 25 nm, (b) 50 nm, and (c) 75 nm Au nanoparticles obtained by numerical simulations and calculations using Eq. (9) indicated in the main text.

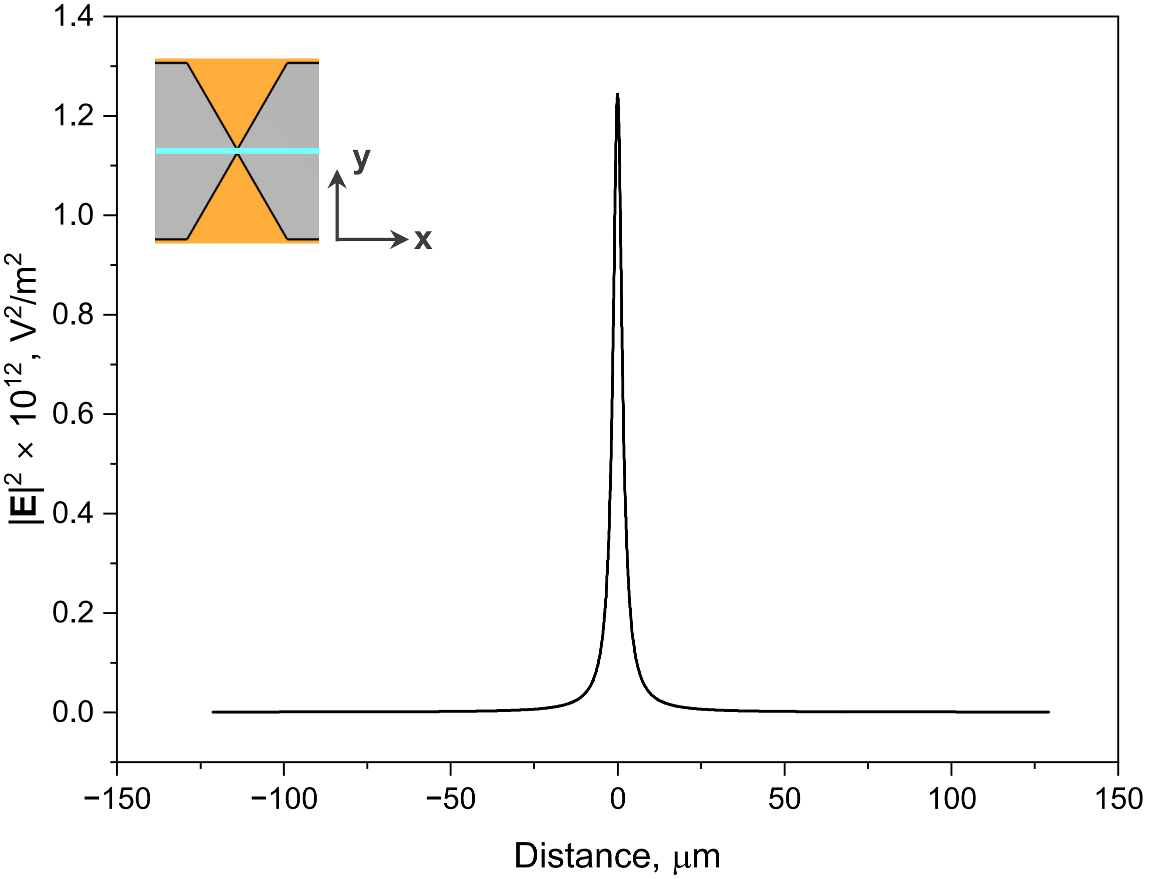

Figure S5. Magnitude of the electric field squared simulated for sawtooth electrode pairs and plotted along the cyan line shown in the inset.

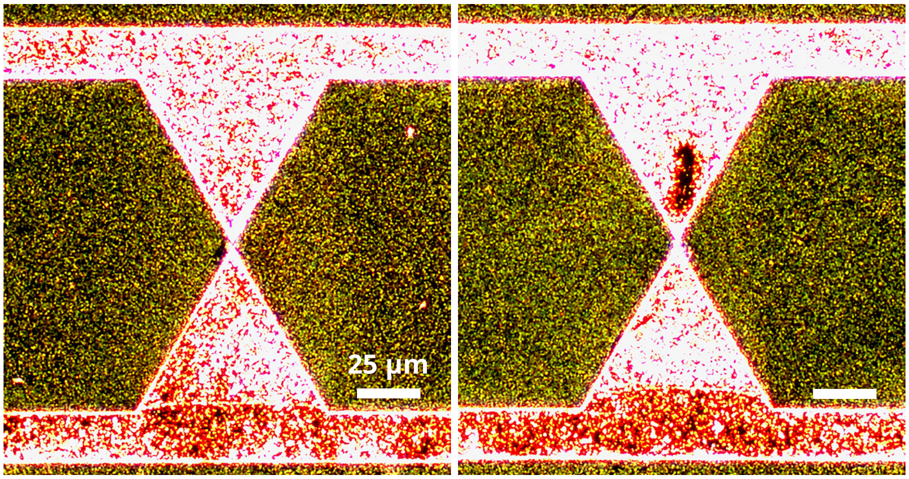

Figure S6. Dark–field images acquired for Au NPs of 50 nm radius trapped near various sawtooth metal electrode pairs after DEP at 15 V and 0.5 MHz performed for 10 min.

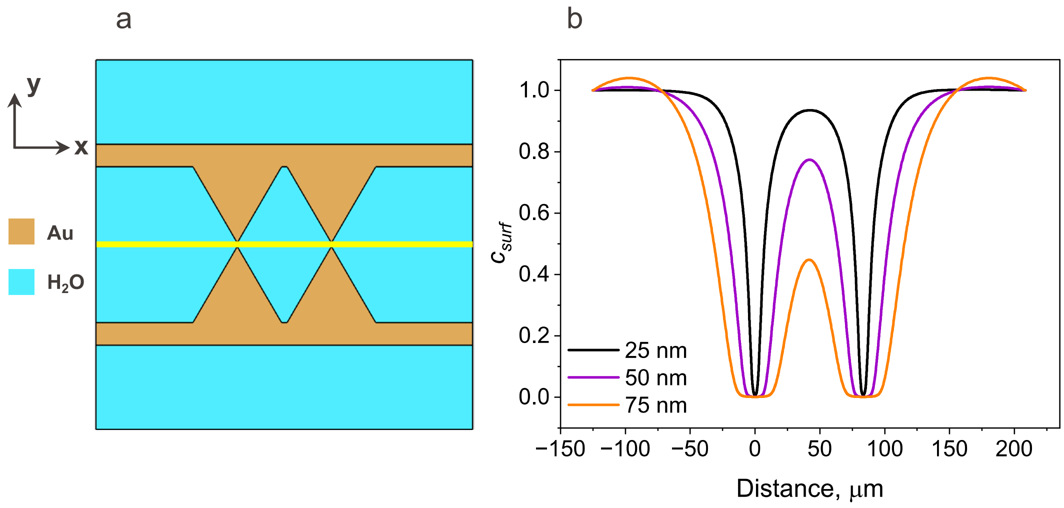

Figure S7. (a) Schematic view of sawtooth electrode pairs with the reduced from 250.4 μm to 83.5 μm intergap distance and (b) simulated concentration profiles plotted along the yellow line in (a).
